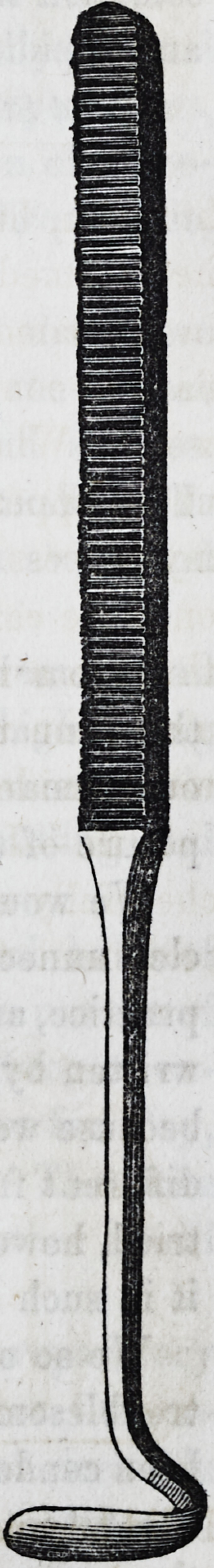# On Fissure of the Hard Palate

**Published:** 1856-10

**Authors:** A. G. Field

**Affiliations:** late Surgeon to the Royal Sea-Bathing Infirmary, and Demonstrator of Anatomy at St. George's Hospital Medical School.


### ARTICLE IX.

*On Fissure of the Hard Palate.* By A. G. Field, F. R. C. S., late Surgeon to the Royal Sea-Bathing Infirmary, and Demonstrator of Anatomy at St. George's Hospital Medical School.

The great difficulty of closing openings in the hard palate, when caused by disease, is admitted by all surgical writers. Even Mr. Fergusson who has done so much to improve the operation of staphyloraphy, while he recommends perseverance in the endeavor, states, that though he has tried the plan of operating suggested by Dr. Warren, of Boston, in several instances, it has always been without success. Mr. Erichsen, in his "Science and Art of Surgery," also states, "This operation has not, I believe, been hitherto successful in this country, and, indeed, been but little practiced, but yet it deserves attention, and holds out a prospect of eventually proving successful in cases of this kind."

The following case not only illustrates the difficulties and disappointments which are liable to attend the operation, but also points out a means by which ultimate success may be attained.

Mrs. G., aged 47, has had disease of the palate and separation of a considerable quantity of bone, on recovery from which an opening was left leading into the nasal fossæ, situated about the middle of the hard palate, and large enough to admit the little finger. The parts have been soundly healed for some months, and she is in good health but experiences much inconvenience from the defect. Her voice is nasal, and it is necessary constantly to wear a plug, to prevent the passage of food and fluids into the nose.

April 15th, 1856.—I tried with the assistance of some medical friends, to produce congelation of the palate, by means of salt and pounded ice, but in this we totally failed, even after more than an hour's perseverance. Our failure is to be accounted for, I suppose, by the high temperature of the parts and the currents of warm air through the mouth and nose, to both of which the freezing mixture would be exposed. Had I succeeded in getting the parts frozen, the advantages to be thereby gained are obvious. All pain would have been saved to the patient, during an operation which might have been accomplished in a very short time, as no interruption would have occurred from hæmorrhage, which gives rise to one of the principal difficulties in plastic operations in the mouth.

Failing in my endeavor to produce congelation I proceeded to operate according to the method of the late Dr. Warren. Having carefully removed the edges of the opening with a small scalpel, an incision of a little more than an inch in length was made, from behind forwards, quite down to the bone on each side, and about half an inch external to the fissure. The soft parts included between these two incisions were then perfectly separated from the bone, by means of an instrument which I will presently describe. When this was done, the flaps so formed by the detached soft structures met together over the opening, where, as soon as bleeding had been arrested by freely bathing with iced water, they were retained by three sutures introduced in the manner practiced by the late Mr. Avery. After the operation was completed one small artery bled freely, and required sustained pressure with the point of a finger to arrest it. The sutures we allowed to remain in five days, during which time the patient abstained from solid food, but partook freely of beef-tea, porter, etc.

The result of this operation was a considerable diminution in the size of the opening, but when the parts had quite cicatrized a hole remained of about half the original size.

June 11.—The operation was repeated, but this time the edges were pared obliquely, the mucous membrane of the nose being removed on one side, that of the palate on the other, so as to allow the edges to overlap each other, whereby a larger extent of raw surfaces were brought into mutual contact. I also used the quilled suture, instead of the simple interrupted suture, which had been employed in the former operation. This interfered less with the circulation in the flaps, while it afforded them more perfect support. After the second operation a hole still remained, about as large as a pin's head. This I tried to close by cauterizing with a hot wire, but as nothing was gained by that, after the cicatrix had formed, I brought the parts together on the 30th of June in the same way as before, in addition to which I supported the flaps in contact by well stuffing the lateral incisions with cotton wool. When the sutures were removed, six days after complete union appeared to have taken place, and I was just rejoicing in my success when I observed a small discharge of mucus, which, on more careful examination, I found had passed from the nose through a minute fissure. In a few days this increased, a little ulceration took place, and at the end of a fortnight the opening was considerably larger than before the last attempt to close it.

17th.—I performed the fourth and last operation, my patient cheerfully submitting, being encouraged by the comfort she experienced from what had already been accomplished. This time I proceeded with, if possible, more elaborate care than on the former occasions, and having completed the other steps of the operation in the same way as before, I carefully dried the parts and washed them over with a solution of gutta percha in chloroform, and then adapted a thin sheet of gutta percha over the whole surface. This I did because I believed failure in my previous efforts to obtain complete union depended on a portion of mucus being drawn from the nose between the edges of the flaps in the act of swallowing, which it is impossible for the patient wholly to avoid. This belief was strengthened by observing, after the failure of the third operation, that the shred of mucus was drawn further into the mouth after each time the patient closed her mouth for the purpose of swallowing the saliva which collected in it, while it was held open for examination; and I imagine it occurs in the following manner:—When the first part of the act of deglutition has been accomplished, and the morsel has passed beyond the reach of the tongue, the upper surface of that organ is left accurately applied to the roof of the mouth, and its return to the position it usually occupies is effected by the genio-hyoglossi muscles, which being inserted near to the median line would, by their contraction, depress the middle of the tongue first, while its sides were still in contact with the palate; by this means a degree of suction would be produced whereby the loose edges of the flaps would be liable to displacement and to have the nasal secretions drawn down between them into the mouth, preventing union of their surfaces, however accurately they may have been adapted. Whether my explanation of the usual cause of failure when this operation has been performed be correct or not, I submit to the decision of more competent judges; but certain it is that I had the happiness of completely accomplishing my object, the removal of a very distressing annoyance to this poor woman, for all that now remains to be seen in her palate is a linear cicatrix in the situation of the opening which had caused her so much trouble, and a broad scar on each side, caused by the gaping of the lateral incisions, which were rapidly filled up with granulations and covered over by mucous membrane.

The instrument which I have found most useful in detaching the soft parts from the hard palate is here represented. It differs from those used by the late Mr.

Avery, in having the blade set on obliquely, so that it allows the flat surface to be applied against the palate, while the handle and hand of the operator are free of the lower jaw. The bend in the neck of the instrument is also advantageous in giving the operator more perfect command over the movements of the blade. With such instruments (a different one being of course necessary for each side) I have always been able to accomplish the most troublesome stage of the operation with ease and rapidity.

28 *Old Steine, Brighton, July* 30, 1856.

## Figures and Tables

**Figure f1:**
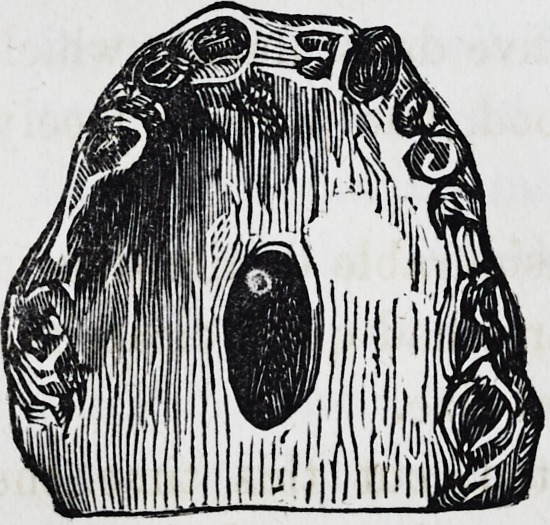


**Figure f2:**
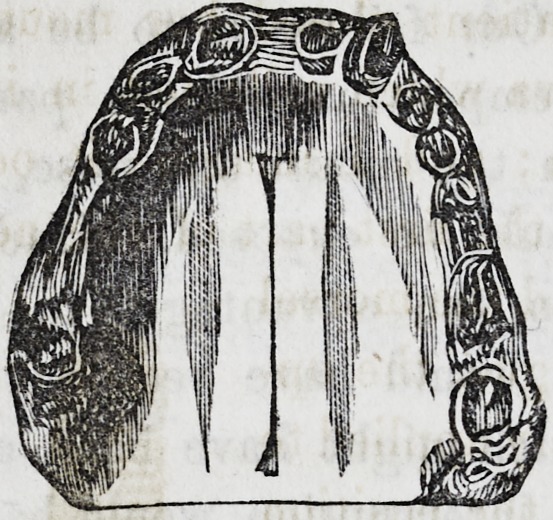


**Figure f3:**
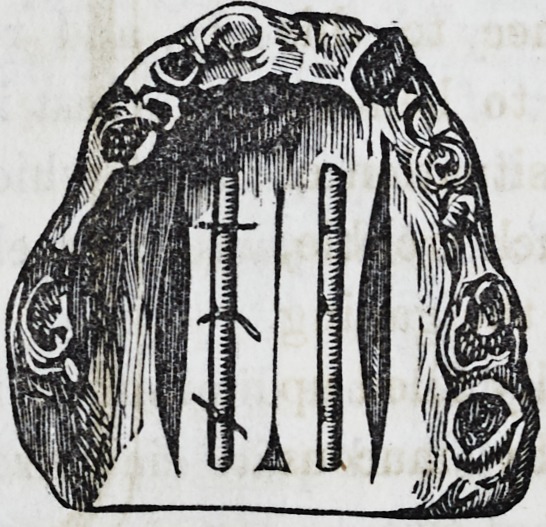


**Figure f4:**